# Green Approach for Fabrication and Applications of Zinc Oxide Nanoparticles

**DOI:** 10.1155/2014/523869

**Published:** 2014-10-14

**Authors:** Brajesh Kumar, Kumari Smita, Luis Cumbal, Alexis Debut

**Affiliations:** Centro de Nanociencia y Nanotecnologia, Universidad de las Fuerzas Armadas-ESPE, Avenida Gral. Rumiñahui s/n, P.O. Box 171-5-231B, Sangolqui, Ecuador

## Abstract

Zinc oxide nanoparticles (ZnO-NPs) are known to be one of the multifunctional inorganic compounds which are widely used in everyday applications. This study aims to fabricate ZnO-NPs using grapefruit (*Citrus paradisi*) peel extract with particle size ranging from 12 to 72 nm. Structural, morphological, and optical properties of the synthesized nanoparticles have been characterized by using UV-Vis spectrophotometer, TEM, DLS, and FTIR analysis. They show the significant photocatalytic degradation efficiency (>56%, 10 mg/L, 6 h) against methylene blue and antioxidant efficacy (≥80% for 1.2 mM) against 1,1-diphenyl-2-picrylhydrazyl. From the results obtained it is suggested that green ZnO-NPs could be used effectively in environmental safety applications and also can address future medical concerns.

## 1. Introduction

In recent years, zinc oxide (ZnO), an important semiconductor with tremendous scientific and technological interest, having a direct wide band gap (3.37 eV, 387 nm, deep violet/borderline ultraviolet (UV)) and a large exciton-binding energy (60 meV) [1], is a highly preferred multitasking metal oxide having a vast list of attractive properties. Due to its unique optical and electrical properties [2, 3], it is regarded as a potential material in optoelectronic applications operating in the visible and near ultraviolet spectral regions. Nanotechnology represents a new promising platform with a broad range of novel uses and permits the controlled synthesis of materials where at least one dimension of the structure is less than 100 nm. Zinc oxide nanoparticles (ZnO-NPs) with the features of large volume to area ratio and high UV absorption have been widely used in many industrial areas such as solar cells [4], UV light-emitting devices [5], gas sensors [6, 7], photocatalysts [8], and pharmaceutical and cosmetic industries [9]. Therefore, the synthesis of ZnO-NPs is of great interest to the researcher.

Several physical and chemical procedures have been used for the synthesis of large quantities of metal nanoparticles in a relatively short period of time. Approaches such as simple solution-based methods, chemical precipitation [10], sol-gel [11], solvothermal/hydrothermal [12, 13], sonochemical [14, 15], electrochemical, and photochemical reduction techniques are most widely used [16]. Chemical methods lead to the presence of some toxic chemicals adsorbed on the surface that may have adverse effects in medical application [17]. Some problems that are often experienced in synthesizing metal nanoparticles are stability and aggregation, control of crystal growth, morphologies, sizes, and distribution, which are important issues and continue to be solved. Thus, there is a need for “green chemistry” that offers numerous benefits of ecofriendliness and compatibility for pharmaceutical and other biomedical applications, where toxic chemicals are not used for the synthesis protocol. The use of agricultural wastes [18] or plants and their parts [19, 20] has emerged as an alternative to chemical synthetic procedures because it does not require elaborate processes such as intracellular synthesis and multiple purification steps or the maintenance of microbial cell cultures [21]. However biosynthesis of zinc oxide nanoparticles (ZnO-NPs) by* Aloe vera* [22],* Bacillus cereus* [23],* Fusarium *spp. [24], and* Medicago sativa* [25], as biological materials, is fully explored.

In the present study, spherical shape ZnO-NPs were synthesized and stabilized using* C. paradisi *outer peel extract by the bioreduction method; these nanoparticles were further investigated as (a) catalyst for the degradation of methylene blue (MB) in the presence of direct sunlight and (b) antioxidant activity using 1,1-diphenyl-2-picrylhydrazyl (DPPH^∙^). Grapefruits (*Citrus paradisi* L.) are unique with sensory quality of sweet and tart taste, having red pigment in the juice vesicles and relatively high levels of bioactive compounds. The major bioactive compounds present in grapefruits include flavonoids, limonoids, carotenoids, furocoumarins, and organic acids [26, 27]. MB is one of the well-known basic/cationic dyes and has been widely used for dying materials for wood, silk, and cotton [28]. The removal of MB from water is very important due to its toxicity.

## 2. Experimental

### 2.1. Materials

All chemicals used were of analytical grade and used without any purification. ZnSO_4_ *·*7H_2_O (>99.0%) and methylene blue (99.5%) were purchased from the Spectrum (USA). DPPH (>99.5%) was purchased from Sigma Aldrich, USA. Milli-Q water was used in all experiments.* C. paradisi* fruit was purchased from the Sangolqui local market, near Universidad de las Fuerzas Armadas-ESPE, Ecuador. The aqueous extract of* C. paradisi* was prepared using thoroughly washed 10 g of yellowish-green outer peel, boiled with 40 mL of double distilled water at 80°C for 30 min. The extract was filtered through muslin cloth and stored in refrigerator at 4°C for further experiments.

### 2.2. Synthesis of Zinc Oxide Nanoparticles

Nanoparticles of ZnO were prepared using the 3 mM ZnSO_4_ *·*7H_2_O (aq) solution. In a typical preparation, 3 mL of the aqueous peel extract of* C. paradisi* was added to 10 mL of 3 mM ZnSO_4_ *·*7H_2_O and pH was adjusted to 8, using 0.1–1 mL* C. paradisi* peel extract. The mixture was stirred for 3 hours at 75–80°C and kept for observation up to change in the coloration. Change of color from yellowish green to light yellow was proof for the formation of ZnO-NPs. The resulting ZnO-NPs were separated out by centrifugation at 6000 rpm for 15 × 2 mins, washed with deionized water (three times), and dried in a hot air oven at 150°C for 1 hour. Finally, the dried ZnO-NPs (3.25 mg) were resuspended in 13 mL deionized water for further characterization.

### 2.3. Characterization

UV-Vis spectra were measured using a spectrophotometer (Thermo Spectronic, GENESYS 8, England, Quartz Cell, path length 10 mm, and graph plotted on the Origin 6.1 program). The particle size distributions of nanoparticles were determined using the HORIBA, Dynamic Light Scattering (DLS) Version LB-550 program. Size and selective area electron diffraction (SAED) pattern of nanoparticles are studied on transmission electron microscopy (TEM) (FEI, TECNAI, G2 Spirit Twin, Holland). Fourier transform infrared (FTIR-ATR) spectra were recorded on a Perkin Elmer (Spectrum Two) spectrophotometer to hypothesized functional groups involved in the synthesis of ZnO-Nps.

### 2.4. Photocatalytic Degradation Study

Nanosized ZnO particle is a good photocatalyst to degrade organic contaminants, such as MB. The photocatalytic degradation of MB using ZnO-NPs is investigated with the following process: four separate sets of experiments were performed to study the decomposition of MB at pH 7 in direct sunlight. In set 1, 5 mL MB (10 mg/L) was taken in a vial and kept in sunlight. In sets 2–4, the synthesized ZnO-NPs (250 *μ*g/mL) were used; 5 mL MB (10 mg/L) and 500, 300, and 100 *μ*L ZnO-NPs were mixed and kept in sunlight. All four sets of reactions were observed after 1, 3, and 6 hrs. Finally, the rate of MB dye decomposition was monitored by taking 4 mL samples from each set and recording the UV-Vis spectra in the wavelength, *λ*_max⁡_ 664 nm, before and after degradation. Photocatalytic degradation percentage of MB was calculated using

(1) $$\begin{array}{c} \eta =\left[1-\frac{A_{t}}{A_{0}}\right]\times 100\text{\%}, \end{array}$$

where *η* is the rate of decomposition of MB in terms of percentage, *A*_0_ is the initial absorbance of MB solution, and *A*_*t*_ is the absorbance of the dyes at time *t*.

### 2.5. Evaluation of Antioxidant Activity

The scavenging activity of the ZnO-NPs was measured by using DPPH^∙^ as a free radical model and a method adapted from Kumar et al. (2014) [29] with slight modification. An aliquot (1000–200 *μ*L) of ZnO-NPs (250 *μ*g/mL) or control and (1000–1800 *μ*L) of H_2_O was mixed with 2.0 mL of 20 *μ*M (DPPH^∙^) in absolute methanol. The mixture was shaken vigorously and allowed to stand at room temperature for 30 min in the dark. Absorbance of the mixture was measured spectrophotometrically at 517 nm, and the free radical scavenging activity was calculated using

(2) Scavenging  effect(%)  =[1−{absorbance  of  sampleabsorbance  of  control}]×100.

The scavenging percentages of all samples were plotted. The final result was expressed as % of DPPH^∙^ free radical scavenging activity (mM).

## 3. Results and discussion

### 3.1. UV-Vis Study

**Figure 1:**
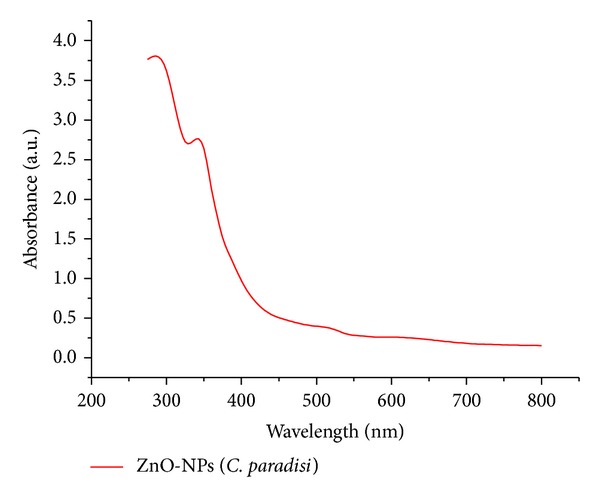
UV-Vis spectra of synthesized ZnO-NPs using* C. paradisi *peel extract.

UV-Vis spectral analysis was used to confirm the formation of ZnO-NPs in the solutions. UV-visible absorption spectra of the ZnO-NPs synthesized from the mixture with aqueous peel extract of* C. paradisi* is presented in Figure 1. It is generally recognized that UV-Vis spectra could be used to examine the size and shape controlled nanoparticles in aqueous suspension [30, 31]. ZnO-NPs exhibit strong UV absorption spectra with the absorption peak ranging from 360 to 375 nm due to their excitonic transition [32].

### 3.2. TEM-SAED Study

**Figure 2:**
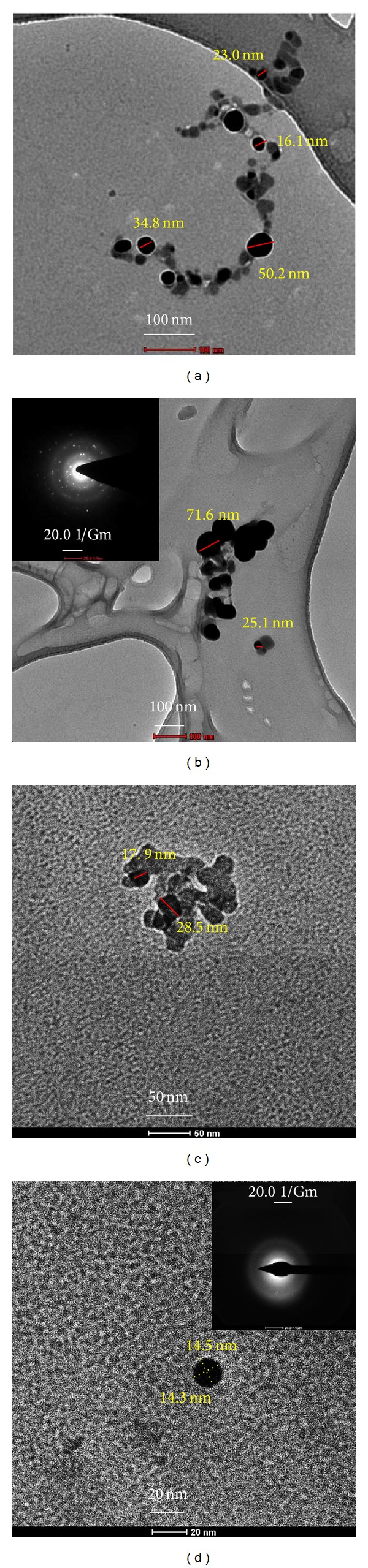
TEM and SAED micrograph of ZnO nanostructures (a–d).

The TEM monographs in Figures 2(a)-2(d) clearly show the distribution of spherical ZnO nanoparticles prepared by* C. paradisi *extract. The ZnO-NPs were homogeneous and agglomerated with a particle size ranging from 12 to 72 nm with some deviations. The diffraction pattern in SAED images shows the crystalline nature of nanoparticles.

### 3.3. DLS Study

**Figure 3:**
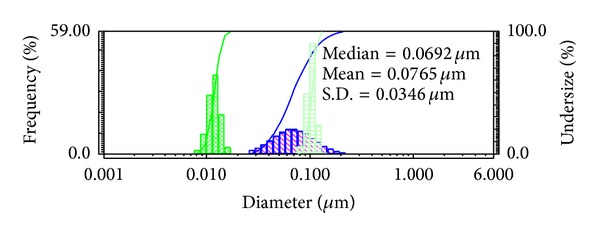
DLS pattern of synthesized ZnO-NPs.

The DLS size distribution image of ZnO-NPs is shown in Figure 3. From the results, the calculated mean particle size distribution of ZnO-NPs is 76.5 nm and the S.D. is 34.6 nm suggesting promising catalytic activity for some specific applications.

### 3.4. FTIR Study

**Figure 4:**
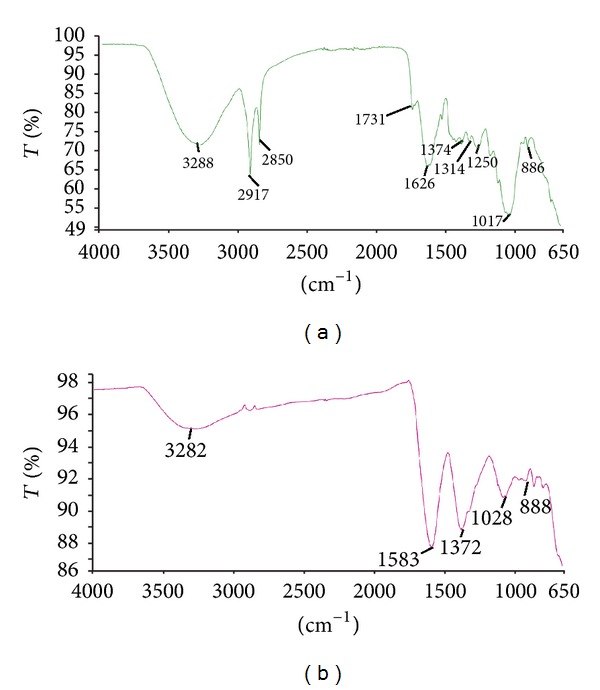
FTIR spectrum of (a)* C. paradisi *peel extract and (b) synthesized ZnO-NPs.

The functional groups present on the surface of* C. paradisi *peel extract and ZnO-NPs were investigated by FTIR analysis (Figures 4(a) and 4(b)) using ATR mode. In Figure 4(a), the broad peak observed at 3288 cm^−1^ indicates the existence of –OH group, whereas the bands centered at 1731 and 1626 cm^−1^ can be assigned to the C=O group. The associated peaks at 3282 cm^−1^ and 1731 cm^−1^ confirm the presence of the free  –COOH. The band observed at 2917 cm^−1^ and 2850 cm^−1^ could be assigned to the C–H stretching vibrations of methyl, methylene, and methoxy groups [20]. The peak observed at around 1017 cm^−1^ indicated C–O stretching and the peak at 1374 cm^−1^ can be attributed to the aromatic C=C bond [22]. The band at 749 cm^−1^ signified the presence of R–CH group. There is a slight deviation of peaks that were observed in Figure 4(b); the bonded hydroxyl groups observed at 3282 cm^−1^, C–O at 1028 cm^−1^ and C=O/C=N group shifted to 1583 cm^−1^. It clearly indicates the involvement of –OH, –COOH, and C=O in the ZnO-NPs synthesis. The peaks in the region below 700 cm^−1^ are allotted to Zn–O [32] and it shows ZnO-NPs absorption band near 650 cm^−1^. Thus, it could be concluded that the ZnO-NPs are synthesized and stabilized by flavonoids, limonoids, and carotenoids molecules that also prevent aggregation.

### 3.5. A Tentative Mechanism for the Formation of ZnO-NPs

**Figure 5:**
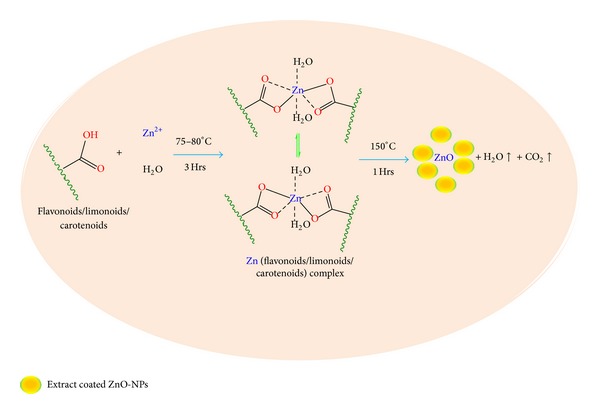
A tentative mechanism for the formation of ZnO-NPs.

Based on our experimental results, we propose the tentative mechanism based on other works [33] for the formation of ZnO-NPs induced by* C. paradisi* peel extracts as shown in Figure 5. The flavonoids/limonoids/carotenoid molecules have free OH/COOH, which can react with ZnSO_4_ to form zinc flavonoids/limonoids/carotenoid complex. After completion of the reaction, the solution was centrifuged and dried in a hot air oven. During drying, conversion of zinc flavonoids/limonoids/carotenoid complex into ZnO-NPs takes place.

### 3.6. Photocatalytic Degradation of Methylene Blue

**Figure 6:**
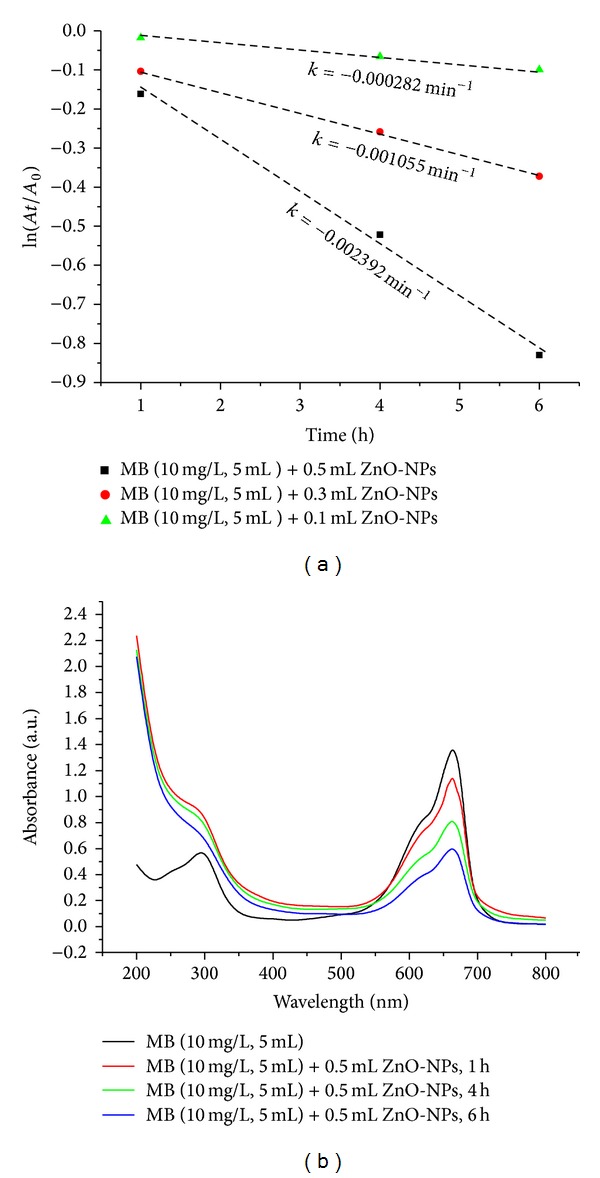
(a) Kinetic plot of ln⁡⁡(*A*_*t*_/*A*_0_) v/s time and (b) optimized photocatalytic pattern of ZnO-NPs for remediation of MB at pH 7.

The rate of MB decomposition catalyzed by ZnO-NPs is assumed to be fitted by a first-order rate law [18, 34] showing a linear relationship between ln⁡⁡(*A*/*A*_0_) and reaction time (Figure 6(a)) and the slope gives the rate constant in each case. The optimal quantity of ZnO-NPs for determining the photocatalytic degradation reaction is 500 *μ*L. When the ZnO nanocatalyst quantity is 100 *μ*L, the rate of photodegradation is very slow and increases with the increasing catalyst concentration. The observed rate constant for 500 *μ*L nanocatalyst is 0.002392 min^−1^. Methylene blue dye shows the prominent peak at 664 nm as shown in Figure 6(b). The peak intensity decreases gradually with the addition of ZnO-NPs under sunlight and shows more than 56% degradation within 6 h. A basic mechanism of photocatalytic reaction on the generation of electron-hole pair and its destination is as follows: when a photocatalyst is illuminated by the light stronger than its band gap energy, electron-hole pairs diffuse out to the surface of the photocatalyst and participate in the chemical reaction with the electron donor and acceptor. Those free electrons and holes transform the surrounding oxygen or water molecules into OH^∙^ free radicals with super strong oxidization [35].

### 3.7. Evaluation of Antioxidant Activity

**Figure 7:**
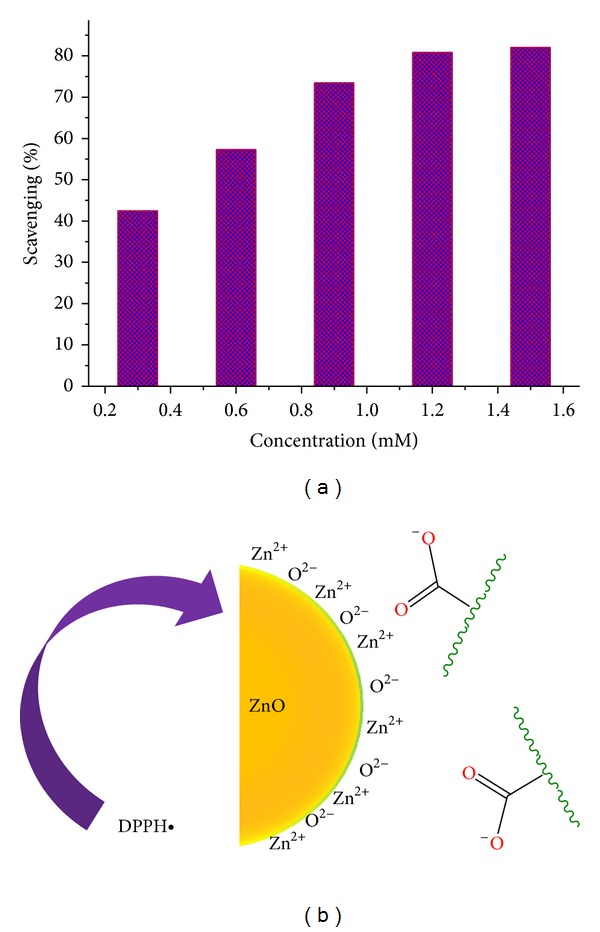
(a) The antioxidant efficacy of ZnO-NPs against DPPH^∙^ and (b) most probable mechanism.

DPPH^∙^ was first used as a preliminary radical scavenging activity test. In the DPPH^∙^ method, the antioxidant reacts with the stable DPPH^∙^ (deep violet color) and converts it into 1,1-diphenyl-2-picrylhydrazine with discoloration (light yellow color). In the present study (Figure 7(a)), the percentage of free radical scavenging activity at different concentrations ranging from 0.3 to 1.5 mM for the ZnO-NPs for DPPH radical was found to increase with concentration and to peak at ≥80% at 1.2 mM. The antioxidant efficacy against DPPH^∙^ is probably derived through the electrostatic attraction between negatively charged bioactive compounds (COO^−^, O^−^) of* C. paradisi* and positively charged nanoparticles (ZnO = Zn^2+^ + O^2−^) as shown in Figure 7(b). ZnO-NPs bound to the phytochemicals and their bioactivity increases synergistically. The effect of activity depends on the site of attachment of the metals and its consequent impact on the activity of the antioxidant agent [29, 36]. Generally, reactive oxygen species (ROS) produced by the* in vitro* methods mentioned above can interact with certain transition metal ions to yield a highly reactive oxidizing species (hydroxyl radicals). This antioxidant reacts further with the stable free radicals available, thereby causing their free radical scavenging activity.

## 4. Conclusions

The current study shows the green approach for fabrication of ZnO nanoparticles and it is responsible for significant photocatalytic and antioxidant activity. It is also believed that* C. paradisi* peel extract promotes the fabrication of the ZnO-NPs with particle size ranging from 12 to 72 nm. The formed ZnO-NPs are highly stable and exhibited more than 56% degradation of MB in sunlight for 6.0 h. In addition, the current study has clearly demonstrated that the ZnO-NPs are responsible for significant antioxidant activity (≥80% for 1.2 mM).
